# Therapeutic repurposing of old drugs to modulate the tumor immune microenvironment and enhance immunotherapy efficacy

**DOI:** 10.1016/j.jpha.2025.101510

**Published:** 2025-12-08

**Authors:** Yakai Song, Nannan Zheng, Xu Yang, Zhaofan Tao, Qinghui Wang, Zhiyue Cao, Yi Zhang, Mengmeng Li, Ruixin Mao, Yuhao Chen, Chen Zhao, Huanjie Yang, Bin Yang, Qiuyue Ma, Liangcan He, Shaoqin Liu, Kai Li

**Affiliations:** a School of Medicine and Health, Harbin Institute of Technology, Harbin, 150001, China; b Zhengzhou Research Institute of Harbin Institute of Technology, Zhengzhou, 450000, China; c School of Life Science and Technology, Harbin Institute of Technology, Harbin, 150001, China; d School of Information Engineering, East University of Heilongjiang, Harbin, 150001, China; e School of Instrumentation Science and Engineering, Harbin Institute of Technology, Harbin, 150001, China

**Keywords:** Drug repositioning, Antitumor, Immunotherapy, Tumor microenvironment, Immunosuppression

## Abstract

Although immunotherapy has demonstrated remarkable progress in cancer treatment, its clinical benefits remain restricted to a subset of patients and specific cancer types, primarily due to the immunosuppressive nature of the tumor microenvironment (TME) in solid tumors. Therefore, many strategies have focused on targeting the immunosuppressive TME to enhance immune-mediated tumor eradication. In parallel, the repositioning of old drugs represents an attractive discovery approach compared with the traditional *de novo* drug discovery process, which is time-consuming and costly. Thus, repurposing US Food and Drug Administration (FDA)-approved old drugs to modulate the tumor immune microenvironment represents a promising strategy to augment the effectiveness of cancer immunotherapy. Indeed, emerging evidence indicates that several approved drugs can reprogram the tumor immune landscape, thereby enhancing responses to immunotherapy. This review provides a comprehensive overview of US FDA-approved old drugs with immunomodulatory properties in the tumor context. We discuss their mechanisms in reversing immunosuppression, summarize key findings from preclinical studies and clinical trials involving their combination with immunotherapies, and outline future perspectives for their clinical translation. Collectively, this work highlights the translational potential of drug repurposing as a strategy to expand the therapeutic reach of cancer immunotherapy.

## Highlights

• Repurposed drugs reprogram the tumor microenvironment to enhance immunotherapy.
• Mechanisms by which repurposed drugs reverse immunosuppression are detailed.
• Preclinical and clinical data on combinations and future directions are summarized.

## Graphical abstract

**Figure:**
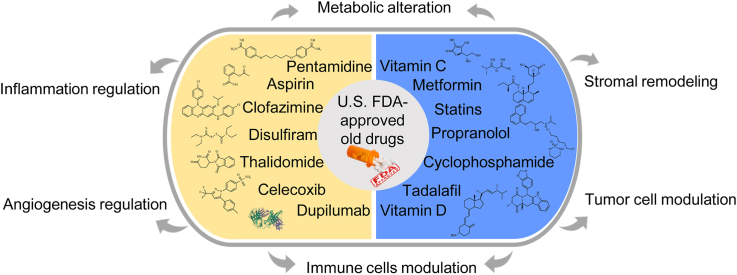

## 1 Introduction

Immunotherapy has emerged as a revolutionary approach in cancer treatment, harnessing the body's immune system to recognize and eliminate cancer cells. It has demonstrated remarkable success in certain malignancies and has transformed the landscape of cancer therapy. Despite this success, only a subset of patients and specific tumor types exhibit durable responses to immunotherapy [1].

A major barrier to the broad success of immunotherapy is the immunosuppressive microenvironment that solid tumors establish to evade immune attack. The tumor microenvironment (TME) comprises various components, including immunosuppressive cells, cytokines, and other molecules that dampen the immune response [2]. Consequently, even in the presence of potent immunotherapeutic agents, the immune system often fails to mount an effective antitumor response.

To overcome these limitations, numerous strategies have been developed to remodel the immunosuppressive microenvironment and enhance the efficacy of immunotherapy [2]. Current research focuses on modulating the TME to restore immune surveillance and strengthen antitumor immunity. These approaches aim to reverse immunosuppression, activate the immune system, and improve the recognition and targeting of cancer cells.

In parallel, the repositioning of US Food and Drug Administration (FDA)-approved old drugs has emerged as an attractive approach to improve cancer control. Compared with traditional *de novo* drug discovery, which is time-consuming and costly, drug repositioning involves repurposing existing US FDA-approved agents for new therapeutic indications. This approach leverages known safety profiles and establishes dosing regimens, potentially accelerating translation to the clinic [3]. Indeed, several reports have shown that certain US FDA-approved old drugs can reprogram the TME to improve the efficacy of chemotherapy or immunotherapy [4]. Therefore, drug repurposing offers a promising strategy for advancing cancer immunotherapy by modulating the TME. This strategy harnesses existing pharmacological knowledge to expedite therapeutic innovation and improve clinical outcomes.

While earlier reviews have outlined the landscape of the TME and drug repurposing, they have primarily focused on constructing overarching frameworks and general strategies rather than systematically addressing how old drugs can be leveraged to enhance the efficacy of immunotherapy [[5], [6], [7]]. Here, we comprehensively review US FDA-approved old drugs with known immunomodulatory activity, highlighting their potential to modulate the complex and dynamic TME, and offer insights into the future prospects for repurposing these agents for cancer immunotherapy. Compared with earlier works [[5], [6], [7]], this review not only expands the drug repertoire but also compiles each compound's preclinical dosing regimens, immunomodulatory mechanisms, and the latest clinical advances in combination with immunotherapy, thereby providing a comprehensive foundation for future mechanistic studies and trial design. Ultimately, we anticipate this review will serve as a reference that facilitates the rational repurposing of these drugs for cancer immunotherapy and accelerates the development of effective, accessible treatment options for patients.

## 2 Overview of the immunosuppressive TME in solid tumors

**Fig. 1:**
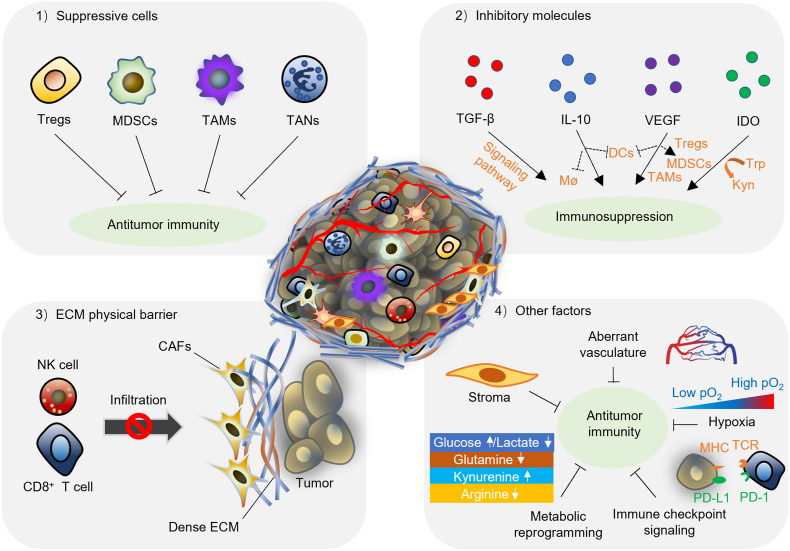
An overview of the immunosuppressive landscape within the solid tumor microenvironment (TME). Solid tumor cells are shielded from immune attack by multiple factors within the TME: 1) suppressive cell types; 2) inhibitory molecules that dampen antitumor responses; 3) the extracellular matrix (ECM) acting as a physical barrier to immune cell infiltration; and 4) other factors such as aberrant vasculature, stroma, metabolic reprogramming, hypoxia, and immune checkpoint signaling that impair the antitumor action of CD8^+^ T cells, natural killer (NK) cells, and dendritic cells (DCs). Tregs: regulatory T cells; MDSCs: myeloid-derived suppressor cells; TAMs: tumor-associated macrophages; TANs: tumor-associated neutrophils; TGF-β: transforming growth factor-beta; IL-10: interleukin-10; VEGF: vascular endothelial growth factor; IDO: indoleamine 2,3-dioxygenase; CAFs: cancer-associated fibroblasts; MHC: major histocompatibility complex; PD-1: programmed cell death protein 1; PD-L1: programmed death ligand 1; pO_2_: partial pressure of oxygen; TCR: T-cell receptor; Kyn: kynurenine; Trp: tryptophan.

In solid tumors, the immunosuppressive microenvironment refers to the conditions and factors within the tumor that hinder the immune system's ability to recognize and eliminate cancer cells effectively. It is characterized by a complex network of immune cells, signaling molecules, and extracellular matrix (ECM) components that collectively create an environment that supports tumor growth and facilitate immune evasion (Fig. 1).

The immunosuppressive nature of the TME is largely driven by specific immune cell populations. Regulatory T cells (Tregs) and myeloid-derived suppressor cells (MDSCs) are major contributors, suppressing antitumor immunity by inhibiting effector T cells and releasing inhibitory factors [8]. Furthermore, distinct subtypes of tumor-associated macrophages (TAMs), tumor-associated neutrophils (TANs), subsets of T helper (Th) cells, and tumor-associated dendritic cells (DCs) also contribute to immune suppression in almost all solid neoplasms [9].

The TME is also characterized by the production of various soluble immunosuppressive factors that further inhibit immune cell function. These include transforming growth factor-beta (TGF-β), interleukin-4 (IL-4), IL-10, and vascular endothelial growth factor (VEGF) [10]. Collectively, these mediators foster a tolerogenic state within the tumor, preventing immune cells from mounting a robust antitumor response.

Additionally, ECM components within the TME contribute to immunosuppression [11]. The ECM is a network of proteins and other macromolecules (including proteoglycans, glycosaminoglycans and glycoproteins) that are primarily secreted by cancer-associated fibroblasts (CAFs). These components provide structural support to tissues and regulate cellular behavior. In solid tumors, the ECM acts as a physical barrier, restricting immune cell infiltration and impairing their function. The dense and fibrotic nature of the ECM limits the delivery of immune cells and therapeutic agents to the tumor site, further impeding the immune response.

In addition, various other factors, including aberrant vasculature, stroma, metabolic reprogramming, hypoxia, and immune checkpoint signaling, contribute to immunosuppression within the TME [12]. Collectively, these factors create a hostile environment that suppresses immune responses and allows cancer cells to evade immune surveillance. Therefore, targeting the TME is crucial for developing effective cancer treatments.

## 3 Strategies to overcome the immunosuppressive TME

**Fig. 2:**
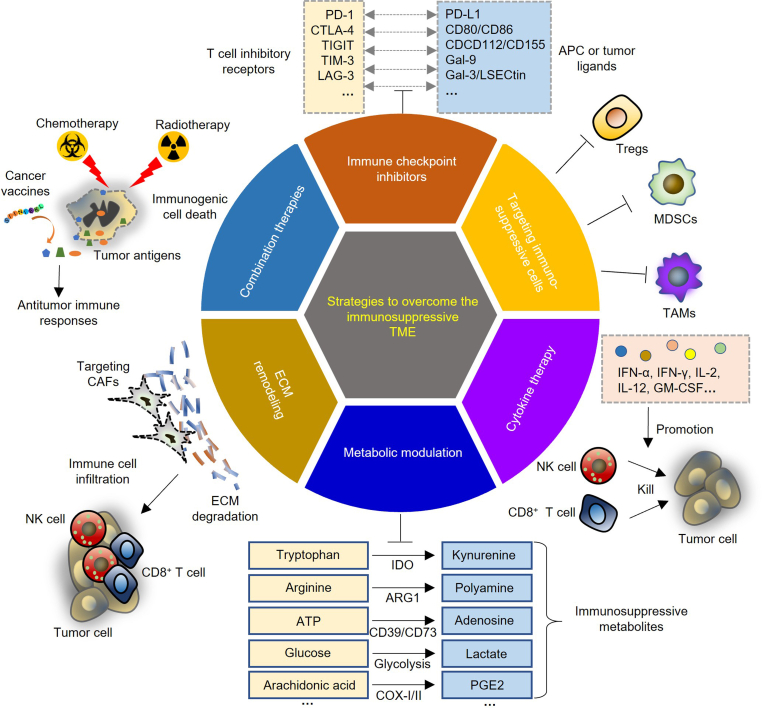
Strategies to overcome the immunosuppressive tumor microenvironment (TME), including checkpoint blockade of programmed cell death protein 1 (PD-1) and cytotoxic T-lymphocyte-associated protein 4 (CTLA-4), targeting immunosuppressive cells, cytokine therapy, metabolic modulation, extracellular matrix (ECM) remodeling, and combination regimens that normalize the TME for effective immunotherapy. APCs: antigen-presenting cells; ARG1: arginase 1; CAFs: cancer-associated fibroblasts; CD39: ectonucleoside triphosphate diphosphohydrolase-1; CD73: ecto-5′-nucleotidase; CD80: cluster of differentiation 80; CD86: cluster of differentiation 86; CD112: nectin-2; CD155: poliovirus receptor; COX: cyclooxygenase; GM-CSF: granulocyte-macrophage colony-stimulating factor; IDO: indoleamine 2,3-dioxygenase; IFN-α: interferon-alpha; IFN-γ: interferon-gamma; IL-2: interleukin-2; IL-12: interleukin-12; LAG-3: lymphocyte activation gene 3; LSECtin: liver and lymph node sinusoidal endothelial cell C-type lectin; NK cells: natural killer cells; PD-L1: programmed death ligand 1; PGE2: prostaglandin E2; TAMs: tumor-associated macrophages; TIGIT: T-cell immunoreceptor with Ig and ITIM domains; TIM-3: T-cell immunoglobulin and mucin domain-containing protein 3; Tregs: regulatory T cells; Gal-9: galectin-9; Gal-3: galectin-3; ATP: adenosine triphosphate.

The immunosuppressive TME within solid tumors presents a significant barrier to effective antitumor immune responses. Therefore, overcoming immunosuppression is a key objective in cancer treatment and involves various approaches aimed at promoting immune activation and restoring immune control over tumor cells. To date, diverse strategies have been employed to overcome immunosuppression in the TME [13], such as immune checkpoint inhibitors, targeting immunosuppressive cells, cytokine therapy, metabolic or ECM modulation, and combination therapies (Fig. 2).

Immune checkpoint inhibitors, such as antibodies targeting programmed cell death protein 1 (PD-1), programmed cell death ligand 1 (PD-L1), and cytotoxic T-lymphocyte-associated protein 4 (CTLA-4), are designed to block inhibitory pathways or immunosuppressive signals that dampen immune responses within the TME. By alleviating these inhibitory constraints, these inhibitors help reinvigorate T-cell activity and promote antitumor immunity. The therapeutic potential of these inhibitors extends beyond the immediate treatment period, as they can also induce durable immunological memory, providing long-term immune surveillance against tumor recurrence [14].

Targeting immunosuppressive cells within the TME represents another promising strategy to restore the antitumor immune response. For instance, approaches targeting Tregs aim to attenuate their suppressive activity, either through depletion or functional inhibition, allowing effector T cells to mount a robust antitumor response [15]. Similarly, MDSCs can be inhibited by blocking their recruitment, differentiation, or inhibitory functions, thereby mitigating their immunosuppressive impact and facilitating enhanced immune responses [16]. Furthermore, targeting TAMs or TANs aims to reprogram their polarization toward an antitumor phenotype, thereby bolstering immune responses [17].

Cytokine therapy enhances antitumor immunity by administering signaling proteins that regulate immune cell activity. Key cytokines, such as interferons (e.g., interferon-α (IFN-α), IFN-γ), interleukins (e.g., IL-2, IL-12), and growth factors (e.g., granulocyte-macrophage colony-stimulating factor (GM-CSF)), are used to stimulate the activation and proliferation of immune cells, thereby augmenting antitumor immune responses. The advantage of this approach lies in its ability to potentiate host antitumor immunity, with generally reduced direct cytotoxicity to normal tissues compared with traditional radiation and chemotherapy. However, cytokine therapy may also lead to significant toxicities, as the overactivation of the immune system can trigger systemic inflammatory reactions [18].

Other approaches targeting immunosuppressive components, such as modulating metabolic characteristics or remodeling the ECM within the TME, can improve antitumor immune responses. For instance, inhibiting metabolic regulators such as indoleamine 2,3-dioxygenase (IDO) or blocking adenosine production can alleviate immune suppression and restore antitumor functions [19]. Similarly, enzymatically degrading ECM components or inhibiting ECM deposition by targeting CAFs can enhance immune cell infiltration and facilitate the entry and activation of cytotoxic lymphocytes, such as CD8^+^ T cells and natural killer (NK) cells.

Moreover, combinatorial strategies, such as integrating immune checkpoint inhibitors with chemotherapy, radiotherapy, or targeted therapies, can synergistically enhance immune responses and improve therapeutic efficacy. For example, chemotherapy may induce immunogenic cell death (ICD), thereby releasing tumor antigens and promoting immune recognition. This facilitates overcoming immunosuppression, and when coupled with immune checkpoint inhibitors, can lead to a more robust and sustained antitumor immune response [20]. Although these approaches have demonstrated promising efficacy, overcoming the complexities of immunosuppression within the TME remains a formidable challenge in cancer treatment.

## 4 Repositioning old drugs for the TME modulation

Translating strategies that counteract the immunosuppressive TME into effective clinical therapies remains a major challenge. The conventional *de novo* drug discovery pipeline is often hampered by extensive timelines, prohibitive costs, and high failure rates, creating a significant gap between preclinical discovery and clinical implementation. In this context, drug repurposing—applying existing, approved therapeutics to new indications—presents a promising and efficient alternative. This strategy leverages established safety and pharmacokinetic data to substantially curtail development costs and shorten the development cycle, offering a pragmatic pathway to accelerate the delivery of novel treatment options.

**Fig. 3:**
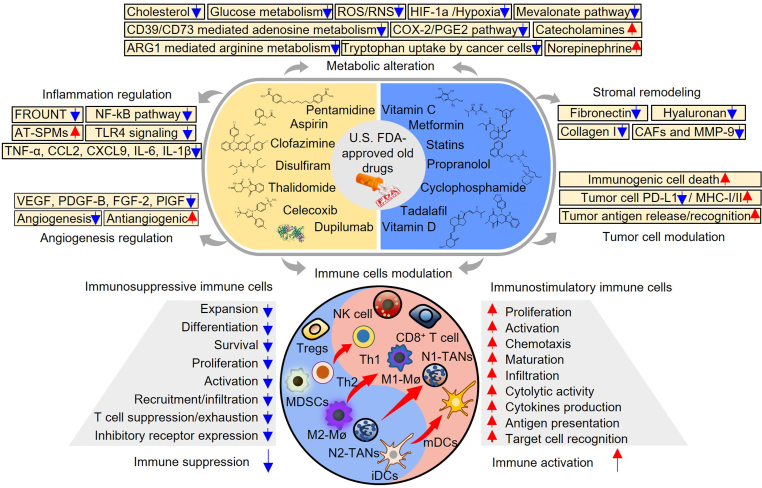
Repositioning old drugs for tumor microenvironment (TME) modulation to boost antitumor immunity. Numerous U.S. Food and Drug Administration (FDA)-approved drugs can reshape the TME through immune cell or tumor cell modulation, metabolic alteration, stromal remodeling, and regulation of inflammation and angiogenesis. ARG1: arginase 1; AT-SPMs: aspirin-triggered specialized pro-resolving mediators; CAFs: cancer-associated fibroblasts; CCL2: C-C motif chemokine ligand 2; CD39: ectonucleoside triphosphate diphosphohydrolase-1; CD73: ecto-5′-nucleotidase; CXCL9: C-X-C motif chemokine ligand 9; COX-2: cyclooxygenase-2; FGF-2: fibroblast growth factor 2; HIF-1α: hypoxia-inducible factor-1-alpha; IDO: indoleamine 2,3-dioxygenase; IL-1β: interleukin-1-beta; IL-6: interleukin-6; iDCs: immature dendritic cells; mDCs: mature dendritic cells; MDSCs: myeloid-derived suppressor cells; Tregs: regulatory T cells; MHC-I/II: major histocompatibility complex class I/II; MMP-9: matrix metalloproteinase 9; NK cell: natural killer cell; N1-TANs: N1-polarized tumor-associated neutrophils; N2-TANs: N2-polarized tumor-associated neutrophils; NF-κB: nuclear factor-kappa B; PDGF-B: platelet-derived growth factor B; PD-L1: programmed death ligand 1; PGE2: prostaglandin E2; PlGF: placental growth factor; RNS: reactive nitrogen species; ROS: reactive oxygen species; TANs: tumor-associated neutrophils; Th1: T helper 1 cells; Th2: T helper 2 cells; TLR4: Toll-like receptor 4; TNF-α: tumor necrosis factor-alpha; VEGF: vascular endothelial growth factor.

Although no drug has yet been formally approved specifically to remodel the TME for immunotherapy, a diverse array of clinically approved pharmaceuticals, originally developed for non-oncological indications, has demonstrated notable antitumor potential. This repertoire of repurposed agents is extensive, spanning multiple therapeutic classes. Examples include anti-inflammatory drugs such as aspirin and celecoxib; cardiovascular agents, including the β-blocker propranolol, lipid-lowering statins, and tadalafil, a phosphodiesterase type 5 (PDE5) inhibitor indicated for erectile dysfunction and benign prostatic hyperplasia; the anti-diabetic agent metformin; and the alkylating agent cyclophosphamide. It further encompasses immunomodulators such as thalidomide and its analogues (originally developed as sedatives), and the anti-allergic antibody dupilumab. Additionally, agents from other therapeutic fields have shown potential, including disulfiram (DSF) for alcohol dependence, the anti-leprosy agent clofazimine, and the antimicrobial pentamidine, as well as the nutritional supplements vitamin C and vitamin D. Collectively, the capacity of these agents to modulate various components of the TME—including immune cells, tumor cells, stroma, metabolism, inflammation, and angiogenesis—suggests they may synergize effectively with existing immunotherapies, thereby offering new strategic avenues to improve cancer treatment outcomes (Fig. 3).

### 4.1 Immune cell modulation

The functional duality of immune cells within the TME encompasses both antitumor and pro-tumor activities. In the early stages of tumorigenesis, antitumor immune populations exhibit the capacity to target and eliminate neoplastic cells. However, malignant cells develop sophisticated mechanisms to circumvent immune surveillance and suppress the cytotoxic functions of antitumor lymphocytes through diverse pathways [21]. Paradoxically, this immune evasion capacity, now recognized as a hallmark of cancer, presents novel therapeutic opportunities to leverage immune cell-mediated antitumor responses. Notably, US FDA-approved repurposed agents demonstrate the potential to remodel the TME via immune cell reprogramming, thereby amplifying antitumor immune responses.

Certain agents enhance antitumor immunity by modulating macrophage polarization in the TME. Celecoxib may inhibit lung tumor growth by modulating the M2/M1 macrophage ratio in the TME [22]. Aspirin can induce the polarization of macrophages toward the M1 phenotype by increasing M1 marker expression while decreasing M2 marker expression, thereby inhibiting cancer cell growth and migration in breast cancer cell lines [23]. Metformin has also been reported to influence macrophage polarization with notable effects on cytokine expression profiles. Ding et al. [24] demonstrated that treating lung cancer cells with metformin *in vitro* resulted in the upregulation of M1-related cytokines and a concomitant decrease in M2-related cytokines. Additionally, Chiang et al. [25] discovered that the AMP-activated protein kinase (AMPK)–nuclear factor κB (NF-κB) signaling pathway, activated by metformin, plays a crucial role in the regulation of genes associated with macrophage polarization, thereby promoting an antitumor phenotype.

The immunosuppressive TME can be reprogrammed by targeting MDSCs and Tregs. Chidamide plus celecoxib (designated CC-01) combined with PD-1 blockade significantly enhances tumor clearance and survival in CT26 models via TME reprogramming—specifically by decreasing populations of Tregs, MDSCs, and TAMs while activating antitumor immunity [26]. Growing evidence indicates that fostering an antitumor immune microenvironment also plays an important role in the antitumor efficacy of propranolol. For instance, propranolol treatment has been reported to lead to a marked reduction in the abundance of intratumoral MDSCs, an increase in T cell infiltration [27], and a reduction in the surgically induced elevation of peripheral Tregs in breast cancer patients undergoing radical mastectomy [28]. Similarly, Tadalafil reduces the accumulation of MDSCs and Tregs in head and neck squamous cell carcinoma (HNSCC) and glioblastoma [29,30]. Finally, vitamin D3 has been shown to sustain immune responses in the early stages of colorectal cancer, thereby modulating Treg function [31].

Certain repurposed agents enhance NK cell functionality and synergize with adaptive immunity to combat tumors. Low-dose aspirin inhibits radiotherapy-induced exosome release in breast cancer, alleviating NK cell suppression [32]. Vitamin C promotes NK cell proliferation without affecting their cytolytic function [33]. Combining propranolol with heat-inactivated 4T1 cells enhances IFN-γ/nitric oxide production and NK cytotoxicity, reduces TGF-β/IL-10 in splenocytes, and ultimately improves survival by suppressing tumor progression [34]. Metformin treatment improves both the cancer surveillance and anticancer activity of NK cells in a p38 mitogen-activated protein kinase (MAPK)-dependent manner. Furthermore, combining metformin with anti-PD-1 antibodies improved the therapeutic response in a B16F10 melanoma model [35]. Statins specifically inhibit hydroxymethylglutaryl coenzyme A reductase (HMGCR), and the downstream depletion of geranylgeranyl pyrophosphate synergizes with IL-2 to activate NK cells [36].

Multiple therapeutic agents enhance T cell efficacy through different mechanisms. Pentamidine acts as a small-molecule PD-L1 antagonist that blocks the PD-1/PD-L1 interaction, augmenting T cell cytotoxicity against cancer cells through increased secretion of IFN-γ, tumor necrosis factor-alpha (TNF-α), perforin, and granzyme B [37]. A combination of celecoxib and intravesical *Bacillus Calmette-Guérin* (BCG) immunotherapy enhances the tumor infiltration of CD4^+^ T cells and improves therapeutic efficacy compared with BCG monotherapy in a murine urothelial cell carcinoma model [38]. Cyclophosphamide reshapes the TME by expanding effector T cells and reducing Tregs [39,40]. DSF directly activates T-cell receptor (TCR) signaling by binding to lymphocyte-specific protein tyrosine kinase (LCK) and enhancing its kinase activity. This activation induces a CD8^+^ T cell immune response and promotes *in vivo* antitumor immunity against both melanoma and colon cancer in mice. Furthermore, this therapeutic effect was potentiated by co-treatment with an anti-PD-1 antibody [41]. Thalidomide and its analogues (e.g., lenalidomide) amplify antitumor immunity by promoting T/NK cell activation and enhancing chimeric antigen receptor T-cell immunotherapy (CAR-T) efficacy against solid tumors [42,43]. Dupilumab, an IL-4R antagonist, reduces circulating monocytes and expands tumor-infiltrating CD8^+^ T cells, synergizing with PD-1/PD-L1 checkpoint blockade in relapsed or refractory non-small cell lung cancer (NSCLC) [44]. Additionally, clofazimine promotes E2F1 activation in CD8^+^ T cells to enhance anti-PD-1/CTLA-4 efficacy while reducing toxicity [45]. Vitamin C has been reported to enhance antitumor immunity by increasing the expression of chemokines and tumor-infiltrating lymphocytes (TILs) [46]. Concurrently, studies have shown that vitamin C can enhance the infiltration of CD8^+^ T cells into the TME and the ability of DCs to stimulate antigen-specific T cell proliferation [47,48]. These strategies collectively illustrate the multifaceted approaches to reprogramming the TME for enhanced immunotherapy.

### 4.2 Tumor cell modulation

Tumor cells act as central regulators of the TME, orchestrating immunosuppression and promoting immune escape through multiple mechanisms. Tumor cells expressing high levels of PD-L1 engage PD-1 receptors on T cells, transmitting inhibitory signals that induce T cell apoptosis and suppress activation [49]. Tumor cells also disrupt CD8^+^ T cell function via metabolic competition, characterized by excessive glucose consumption and lactate secretion (the Warburg effect) [50]. Additionally, tumor cells downregulate major histocompatibility complex (MHC) class I and II molecules through epigenetic modifications and genetic mutations, thereby impairing the presentation of tumor antigens to CD8^+^ T cells and enabling escape from T cell-mediated cytotoxicity [51,52]. Furthermore, tumor cells can directly compromise T cell viability by expressing death ligands such as FasL/TRAIL or releasing reactive oxygen species (ROS), further weakening antitumor immunity [53,54]. Notably, several repurposed drugs can remodel the TME by regulating the expression of PD-L1 and MHC molecules in tumor cells.

Aspirin can significantly decrease PD-L1 expression in lung cancer cells and PD-1 expression in macrophages and CD8^+^ T cells, an effect beneficial for antitumor immunotherapy [55]. Indeed, in a large retrospective chart review of 500 NSCLC patients receiving PD-1 or PD-L1-directed immunotherapy, concomitant daily use of aspirin with anti-PD-L1 therapy was associated with improved clinical outcomes, and the adjuvant use of aspirin exhibited a stronger association with survival in NSCLC patients with low tumoral PD-L1 expression [56]. Yamaguchi et al. [57] demonstrated that the combination of an anti-PD-1 antibody and celecoxib synergistically exerts antitumor effects in a malignant glioma model through the downregulation of PD-L1, which was associated with post-transcriptional regulation of the co-chaperone FK506-binding protein 5 (FKBP5). Fujiwara et al. [58] reported that pomalidomide can inhibit PD-L1 expression on tumor cells to promote cytotoxic T-lymphocyte (CTL) activity *in vitro* and suppress PD-L1 upregulation on antigen-presenting cells (APCs) to prevent peptide-induced T-cell tolerance. Falcinelli et al. [59] showed that propranolol can decrease IFN-γ production and IFN-γ-mediated PD-L1 overexpression in tumor cells. The combination of propranolol and a PD-L1 inhibitor significantly decreased stress-induced metastasis and improved antitumor immunity in ovarian cancer. Statins can increase the expression of MHC class I-related chain A (MICA) in melanoma, thereby facilitating targeting by NK cells [60].

Conversely, certain strategies exploit the upregulation of PD-L1 to sensitize tumors to anti-PD-1 therapy. For instance, Zheng et al. [61] demonstrated that DSF inhibits the activity and expression of DNA methyltransferase 1 (DNMT1), leading to hypomethylation of the interferon regulatory factor 7 (IRF7) gene. This, in turn, upregulates PD-L1 expression and modulates the TME. Paradoxically, while elevated PD-L1 is a marker of immunosuppression, its induction by DSF was found to sensitize tumors to anti-PD-1 therapy, thereby enhancing its therapeutic efficacy in a 4T1 breast cancer mouse model. Furthermore, *in vitro* and *in vivo* studies by Zhou et al. [62] revealed that DSF/Cu^2+^ inhibits poly ADP-ribose polymerase 1 (PARP1), which suppresses glycogen synthase kinase 3β (GSK3β) activity and leads to the upregulation of PD-L1 expression. Consequently, combination therapy with DSF/Cu^2+^ and an anti-PD-1 antibody demonstrated significantly greater antitumor efficacy than either monotherapy in hepatocellular carcinoma (HCC). We hypothesize that the disparate outcomes across studies are attributable to variations in copper bioavailability and tumor-specific contexts. The copper homeostasis within a given tumor type is the primary determinant of whether DSF functions as a direct immune activator or an immune checkpoint blockade (ICB) sensitizer.

### 4.3 Stromal remodeling

Stromal cells within the TME play critical roles in tumorigenesis and progression. Excessive deposition of ECM components, such as collagens I and IV, and hyaluronan (HA), forms a dense physical barrier that impedes immune cell infiltration. Furthermore, activated CAFs secrete immunosuppressive factors, including TGF-β, IL-6, and chemokine (C-X-C motif) ligand 12 (CXCL12) [63]. Aberrant expression of matrix metalloproteinases (MMPs) further contributes to tumor metastasis by promoting proteolytic degradation of ECM components [64].

Metformin has been shown to modulate the ECM and CAFs within the TME. Specifically, Shen et al. [65] reported that metformin treatment in renal fibroblasts effectively impeded angiotensin II (Ang II)-mediated overproduction and accumulation of ECM proteins, notably fibronectin and collagen I. Furthermore, Incio et al. [66] observed that metformin attenuated desmoplasia in preclinical pancreatic cancer models by reducing ECM components, particularly collagen I and HA. Similarly, Hwang et al. [67] demonstrated that the metformin-mediated inhibition of phorbol-12-myristate-13-acetate (PMA)-induced MMP-9 expression contributes to its antimetastatic properties in human fibrosarcoma cells. Vitamin D has been extensively studied for its potential anticancer properties, with evidence indicating its ability to inhibit cancer cell proliferation, induce apoptosis, suppress angiogenesis, and attenuate the protumorigenic functions of CAFs [68]. For instance, the active vitamin D metabolite 1α, 25-dihydroxyvitamin D_3_ (1,25(OH)_2_D_3_) reprograms human colon CAFs into a less activated phenotype, thereby reducing their capacity to remodel the ECM and enhance colon carcinoma cell migration [69]. Moreover, 1,25(OH)_2_D_3_ regulates over a thousand genes in colon CAFs involved in cell adhesion, differentiation, migration, tissue remodeling, blood vessel development, and inflammatory responses. Consistent with these findings, 1,25(OH)_2_D_3_ downregulates miR-10a-5p levels in exosomes secreted by human pancreatic CAFs, thereby mitigating their promigratory and pro-invasive effects on pancreatic carcinoma cells [70].

### 4.4 Metabolic alteration

The establishment of the TME is closely linked to metabolic reprogramming, whereby tumor cells actively suppress antitumor immune responses by altering the local metabolic milieu [71]. Specifically, tumor cells upregulate glycolysis [[72], [73], [74]] and competitively consume nutrients such as glucose and glutamine in the microenvironment, thereby severely compromising antitumor immunity [75]. Concurrently, the accumulation of lactate and tryptophan metabolites (e.g., kynurenine) produced during tumor metabolism can directly inhibit the function of effector immune cells, including NK cells [76] and CD8^+^ T cells, and promote the expansion of immunosuppressive cells such as Tregs and MDSCs [77]. In addition, the hypoxia-induced hypoxia-inducible factor-1-alpha (HIF-1α) signaling pathway can further drive tumor glycolysis, exacerbating the immunosuppressive microenvironment [78]. Therapeutic strategies targeting these key metabolic nodes—such as inhibiting metabolic enzymes like indoleamine 2,3-dioxygenase 1 (IDO1) and arginase-1 (ARG1), or blocking the adenosine signaling pathway—hold promise for reversing immune suppression and improving the efficacy of cancer immunotherapy [79].

Aspirin enhances antitumor immunity by modulating distinct metabolic pathways. For instance, Lei et al. [80] demonstrated that aspirin remodels tumor metabolism by downregulating c-Myc oncoprotein levels, thereby suppressing the expression of the glucose transporter 3 (GLUT3) and key glycolytic enzymes—hexokinase 2 (HK2), muscle-type phosphofructokinase (PFKM), pyruvate kinase M2 (PKM2), and lactate dehydrogenase A (LDHA). In the CT26 tumor model, this metabolic shift synergistically enhanced the efficacy of immune checkpoint blockade (anti-PD-1 and anti-CTLA-4) in a manner critically dependent on CD8^+^ T cells. Notably, this immune-activating potential was further highlighted when aspirin-treated tumor cells, loaded with tumor antigens such as the AH1 or A5 peptide, functioned as a potent therapeutic vaccine that elicited a superior antitumor T-cell response capable of promoting tumor eradication. Furthermore, Cui et al. [81] showed that blocking arachidonic acid (AA) metabolism with aspirin enhanced antitumor immune responses by activating CD8^+^ T cells and inhibiting vasculogenic mimicry. This mechanism also synergized with immune checkpoint inhibitors to improve therapeutic outcomes, particularly in AT-rich interactive domain-containing protein 1A (ARID1A)-deficient colorectal cancer. Ma et al. [82] developed an oral dextran-aspirin nanomedicine (P3C-Asp) that released salicylic acid, scavenged ROS, and modulated gut microbiota via dextran. In primary colorectal cancer (CRC) models, P3C-Asp not only enhanced microbial homeostasis and anticancer immunity but also achieved a 2.1-fold higher tumor suppression rate than aspirin. When combined with an anti-PD-L1 antibody, it further reduced tumor burden and prolonged survival. In a parallel metabolic context, Huang et al. [83] reported that metformin can reprogram tryptophan metabolism, thereby reducing tryptophan uptake by colorectal cancer cells, restoring tryptophan availability to CD8^+^ T cells and enhancing their cytotoxicity. Finally, vitamin C (ascorbic acid), beyond its role as an essential nutrient in biological processes such as collagen synthesis and antioxidant defense, has garnered significant attention for its potential anticancer effects. The antioxidant properties of vitamin C have been hypothesized to protect against cancer by mitigating oxidative stress and DNA damage [84]. Conversely, high-dose vitamin C therapy has been explored as a direct cytotoxic agent against cancer cells through the generation of hydrogen peroxide and oxidative stress within the TME [85].

### 4.5 Inflammation regulation

Chronic inflammation promotes the release of pro-inflammatory mediators, such as IL-6 and prostaglandin E2 (PGE2), which recruit and activate MDSCs and Tregs, thereby establishing an immunosuppressive TME. MDSCs inhibit the function of cytotoxic T cells and NK cells through arginase-1 and inducible nitric oxide synthase (iNOS), while Tregs suppress effector T cell activity through IL-10 and TGF-β. Concurrently, the upregulation of immune checkpoint pathways (PD-1/PD-L1, CTLA-4) further weakens antitumor immunity. Inflammation-induced oxidative stress and epigenetic modifications can cause DNA damage and enhance tumor stemness [86]. Therapeutic strategies targeting these mechanisms, including cyclooxygenase-2 (COX-2) inhibition, MDSC/Treg depletion, and checkpoint blockade, hold promise for reversing immunosuppression and enhancing the antitumor immune response.

Aspirin promotes macrophage clearance of therapy-generated tumor cell debris and inhibits macrophage-secreted proinflammatory cytokines [87]. Similarly, DSF can also regulate the production of several cytokines. For instance, Terashima et al. [88] demonstrated that DSF, a potent inhibitor of the chemokine signal regulator FROUNT, suppresses pro-tumoral macrophage activity and delays tumor progression by disrupting the interaction between FROUNT and chemokine receptors. Moreover, DSF synergizes with anti-PD-1 immune checkpoint inhibitors to increase the infiltration of cytotoxic CD8^+^ T cells, thereby enhancing the antitumor immune response. In a separate study, Hu et al. [89] reported that co-administering antibiotics and copper synergistically enhances DSF's antitumor efficacy. This combination therapy exerts its effect by significantly suppressing the hyperactivated Toll-like receptor 4 (TLR4)/NF-κB signaling pathway and its downstream pro-inflammatory cytokines (e.g., IL-1β, IL-6, and TNF-α), representing a distinct therapeutic mechanism. However, the anticancer mechanism of clofazimine remains controversial; studies suggest that clofazimine acts as an inhibitor of the Wnt signaling pathway [90], a peroxisome proliferator-activated receptor γ (PPARγ) agonist [91], and an anti-inflammatory agent via the inhibition of the Na^+^, K^+^-ATPase and the Kv1.3 potassium channel [92]. Collectively, these properties may contribute to its anticancer potential. Although the complete antitumor mechanisms of pentamidine remain to be fully elucidated, they are thought to involve multiple pathways, such as the phosphatase and tensin homolog (PTEN)-phosphoinositide 3-kinase (PI3K)/protein kinase B (AKT) signaling axis, mitochondrial targeting, and immunomodulatory activities [93]. For example, its immunomodulatory role was highlighted by Seguella et al. [94], who reported that pentamidine, as an S100B inhibitor, exerts a dual antitumor effect. It suppresses S100B-mediated pro-inflammatory activity and disrupts the S100B-wtp53 interaction to restore wtp53-mediated apoptosis. This combined anti-inflammatory and pro-apoptotic action underscores its therapeutic potential for treating colon cancer.

### 4.6 Angiogenesis regulation

Tumor-associated angiogenesis orchestrates the immunosuppressive TME. Hyperactivation of VEGF signaling not only leads to pathological angiogenesis and resultant hypoxia, but also impairs dendritic cell maturation and T-cell infiltration, thereby compromising the antitumor immune response [95]. In addition, aberrant vasculature promotes the accumulation of MDSCs via the VEGF signaling pathway, further suppressing T cell function [96]. Moreover, hypoxia induces the polarization of TAMs into the immunosuppressive M2 phenotype and recruits Tregs, further impairing immune clearance [97]. Together, these mechanisms contribute to the establishment of an immunosuppressive TME, enabling tumors to evade immune surveillance and facilitating tumor growth and metastasis.

Thalidomide can inhibit angiogenesis by reducing the secretion of angiogenic factors such as VEGF and fibroblast growth factor (FGF), which in turn suppresses tumor metastasis and malignant transformation [98,99]. Moreover, Shen et al. [100] demonstrated that thalidomide remodels the aberrant tumor vasculature into a more normalized state by restricting excessive sprouting, increasing pericyte coverage, tightening endothelial junctions, and enhancing perfusion, thereby improving the delivery and efficacy of chemotherapeutic agents. Mechanistically, these effects were partly attributed to thalidomide's ability to restore the balance between pro- and anti-angiogenic factors. Metformin shifts TAM polarization from an M2-like to an M1-like phenotype, thereby impeding tumor growth and angiogenesis *in vivo* [101]. Furthermore, metformin attenuates angiogenesis through the downregulation of VEGF and the suppression of HIF-1α-induced angiogenesis-associated factors, such as platelet-derived growth factor B (PDGF-B), fibroblast growth factor 2 (FGF-2), and placental growth factor (PlGF) [102].

## 5 Future perspectives and challenges

Repurposing approved non-oncological agents to remodel the TME offers a context-specific and potentially accelerated avenue to expand the benefits of immunotherapy. Table 1 [[22], [23], [24], [25], [26], [27],^29^,^31^,^32^,[34], [35], [36], [37], [38], [39],^41^,[43], [44], [45], [46], [47], [48],^55^,[57], [58], [59], [60], [61], [62],^66^,^67^,^69^,^70^,[80], [81], [82], [83],[87], [88], [89],[99], [100], [101]] details selected repurposed drugs that modulate the TME and enhance therapeutic efficacy. Emerging evidence from ongoing clinical trials (Table 2) supports this translational potential; however, translating these findings into reproducible clinical benefit remains challenging and will require coordinated advances across pharmacology, drug delivery, biomarker development, organoid and immune modeling, spatial and computational biology, and trial methodology.

**Table 1:** Repositioning old drugs for tumor microenvironment modulation.

Classification of antitumor Immunity	Drugs	Monotherapy or combination therapy	Tumor model	Dosing regimen	Mechanisms of antitumor immunity	Refs.
Immune cell modulation	Celecoxib	IFN-γ	Murine LLC-1 tumor model	IFN-γ (10000 IU in 200 μL saline, intraperitoneally, once a day for 5 days, discontinued 2 days, then continued 5 days) and celecoxib (60 mg/kg in 200 μL saline, gavage, every other day)	M2/M1 macrophage ratio ↓	[22]
	Aspirin	–	4T1 cells	Aspirin (0.5−2 mM)	M2/M1 macrophage ratio ↓	[23]
	Metformin	–	Murine LLC tumor model	Metformin (100 mg/kg, daily, 21 days, intraperitoneally)	M2-like polarization of macrophages ↓	[24]
		–	Murine MDA-MB231 tumor model	Metformin (200 mg/kg, intraperitoneally, three times a week)	M2/M1 macrophage ratio ↓	[25]
	Celecoxib	Anti-PD-1 antibody, chidamide	Murine CT26 tumor model	Chidamide-k30 and celecoxib (50 mg/kg, orally, daily, days 10–25) and anti-PD-1 antibody (2.5 mg/kg, intraperitoneally, every 3 days, days 10, 13, 16, 19, 22, 25)	Populations of Tregs, MDSCs and TAMs ↓	[26]
	Propranolol	Anti-CTLA4 antibody	Murine MCA205 and MC38 tumor model	Propranolol (0.5 g/L, drinking water) and anti-CTLA4 antibody (200 μg/mouse, intraperitoneally, days 1,3,6)	T cell infiltration ↑	[27]
					MDSCs infiltration ↓	
	Tadalafil	–	HNSCC patients	Tadalafil (10−20 mg/day, ≥20 days)	The number of MDSCs and Tregs ↓	[29]
	Vitamin D	–	Colorectal cancer patients	Vitamin D_3_ (8000 IU/day, 3 months)	The level of Treg ↑	[31]
	Aspirin	Radiotherapy	Murine BT-549 tumor model	Aspirin (25 mg/kg/day, gavage) and total body irradiation (0.1 Gy/min, 10 min, twice a week)	Inhibition of NK cell proliferation ↓	[32]
	Propranolol	Heated 4T1 cells	Murine 4T1 tumor model	Heated 4T1 cell extract immunization (10^6^ cells) and propranolol (6 mg/kg), twice immunized at a one-week interval subcutaneously	Cytotoxicity of NK cells ↑	[34]
	Metformin	Anti-PD-1 therapy	Murine B16F10 tumor model	Anti-PD-1 (0.2 mg, every 3 days, from day 3) and metformin (100 mg/kg, daily) intraperitoneally	NK activity ↑	[35]
	Statins	–	PBMCs	Statins (5 μM)	NK cell activation ↑	[36]
	Pentamidine	–	PD-L1 humanized syngeneic mouse model (4T1, MC38, KLN205, and B16F10)	Pentamidine (10 mg/kg, intraperitoneally, daily)	T-cell-mediated cytotoxicity ↑	[37]
	Celecoxib	BCG	Murine urothelial cell carcinoma model	BCG (instillation, three times a week for 2 weeks) and celecoxib (10 or 100 mg/kg, subcutaneously, twice a day for 21 days)	Tumor infiltration of CD4^+^ T cells ↑	[38]
	Cyclophosphamide	–	Metastatic colorectal cancer patients	Cyclophosphamide (50 mg, orally, twice daily, days 1–7 and 15−21)	Antitumor T Cell response ↑	[39]
	DSF	Anti-PD-1 antibody	Murine B16F10 tumor model	DSF (50 mg/kg, orally, daily) and anti-PD-1 antibody (200 μg, intraperitoneally, day 0, 4 and then every 3rd day)	CD8^+^ T cell immune response ↑	[41]
	Lenalidomide	Anti-PD-1 antibody	Murine MC38-OVA and B16F10-OVA tumor model	Lenalidomide (50 mg/kg, orally, daily) and anti-PD-1 antibody (5 mg/kg, intraperitoneally, every other day)	CD8^+^ T cell activity ↑	[43]
	Dupilumab	PD-(L)1 checkpoint blockade	NSCLC patients	Dupilumab (600 mg, then 300 mg every 3 weeks for 3 treatments, subcutaneously) and continued PD-(L)1 blockade	CD8^+^ T cells ↑	[44]
	Clofazimine	Anti-PD-1 and anti-CTLA-4 antibodies	Murine MC38, D4M.3A, A20 and LL/2 tumor model	Anti-PD-1 antibody and anti-CTLA-4 antibody (100 μg, every 3 days, 5−10 doses), clofazimine (8 mg/kg, every 2 days, 7−12 doses) intraperitoneally	E2F1 activation in CD8^+^ T cell ↑	[45]
	Vitamin C	Anti-PD-L1 antibody	Murine B16-OVA tumor model	Anti-PD-L1 antibody (200 μg, 3 times per week for 2 weeks) and sodium ascorbate (4 g/kg, daily) intraperitoneally	Chemokine and TILs ↑	[46]
		–	Murine 4 T1 tumor model	Vitamin C (2 g/kg/day or 4 g/kg/day, intraperitoneally)	CD8^+^ T cells infiltration and function ↑	[47]
		–	PBMCs	Vitamin C (500 μM)	DC stimulation of autologous antigen-specific T cell proliferation ↑	[48]
Tumor cell modulation	Aspirin	–	A549 and H1299 cells	Aspirin (2.5−5 mM)	PD-L1 ↓	[55]
	Celecoxib	Anti-PD-1 antibody	Murine tumor model with glioma stem cells	Anti-PD-1 antibody (20 mg/kg, day 0; 10 mg/kg every 6 days) and celecoxib (10 mg/kg, daily for 30 days) intraperitoneally	PD-L1 ↓	[57]
	Pomalidomide	4-1BB antibody or OVA vaccine	Murine MC38 or B16-OVA tumor model	4-1BB (200 μg, days 10, 13, 15, 17) and pomalidomide (50 mg/kg, days 10, 13, 15, 17) intraperitoneally; OVA vaccine (0.5 mg, day 6) and pomalidomide (50 mg/kg, days 6, 8, 10, 12) intraperitoneally	PD-L1 ↓	[58]
	Propranolol	PD-L1 inhibitor	Murine ID8 tumor model	Propranolol (2 mg/kg/day, osmotic pump) and PD-L1 inhibitor (200 μg/mouse, 3 times a week for 2 weeks, intraperitoneally)	PD-L1 ↓	[59]
	Statins	–	Murine melanoma model	LB1319-MEL, LB2033-MEL or LB583-MEL (pretreated with 5 μM atorvastatin for 48 h) injected subcutaneously to mice	MICA expression ↑	[60]
	DSF	Anti-PD-1 antibody	Murine 4T1 tumor model	DSF (100 mg/kg, intragastrically, daily) and anti-PD-1 antibody (2 mg/kg, intraperitoneally, days 7, 9, 11, 13)	PD-L1 ↑	[61]
		Anti-PD-1 antibody	Murine Hepa1-6 tumor model	DSF plus copper gluconate (50 mg/kg DSF; 0.15 mg/kg Cu^2+^, orally, daily) and anti-PD-1 antibody (250 μg, intraperitoneally, every 3 days)	PD-L1 ↑	[62]
Stromal remodeling	Metformin	–	Murine AK4.4 tumor model	Metformin (300 mg/kg, drinking water, 2 weeks)	ECM components (collagen I and HA) ↓	[66]
		–	HT-1080 cells	Metformin (5 mM)	MMP-9 ↓	[67]
	Vitamin D	–	Primary human colon normal and tumor fibroblasts	1,25(OH)_2_D_3_ (100 nM)	The protumoural activation of CAFs ↓	[69]
		–	Pancreatic cancer cells and CAFs	–	Mir-10a-5p levels ↓	[70]
Metabolic alteration	Aspirin	AH1 or A5 peptide	Murine CT26 tumor model	CT26 cells (aspirin pretreatment) and indicated peptide (100 μg) subcutaneous inoculation twice	GLUT3 and glycolytic enzymes ↓	[80]
					Antitumor T cell response ↑	
		Anti-PD-1 antibody	ARID1A-deficient humanized PDX mouse model	Aspirin (600 mg/mL, drinking water, daily) and anti-PD-1 antibody (10 mg/kg, every 3 days for 2 weeks)	AA metabolism ↓	[81]
		Anti-PD-L1 antibody	Murine CT26 tumor model	P3C-Asp (35 mg aspirin/kg, days 0, 2, 4, 6) and anti-PD-L1 (100 μg/mouse, days 1, 4, 7) intraperitoneally	ROS ↓	[82]
	Metformin	–	Murine APC^min/+^ colorectal cancer model	Metformin (250 mg/kg/day, gavage)	Tryptophan metabolism in colorectal cancer cells ↓	[83]
					Tryptophan metabolism in CD8^+^ T cells ↑	
Inflammation regulation	Aspirin	–	Murine Lewis lung carcinoma (LLC) tumor model	Aspirin (30 mg/kg/day via oral gavage)	Proinflammatory cytokine ↓	[87]
	DSF	Anti-PD-1 antibody	Murine LLC and B16 tumor model	DSF (0.8 mg DSF/1 g CE-2 powder diet, orally, daily) and anti-PD-1 antibody (200 μg, days 5, 8, 14, 18, intraperitoneally)	Chemokine-mediated FROUNT functions ↓	[88]
		Antibiotics and copper	Murine B16F10 tumor model	Antibiotics (drinking water, 2 weeks, until end of experiment) and DSF plus copper gluconate (50 mg/kg DSF; 0.15 mg/kg Cu^2+^, day 7 post-tumor inoculations)	Pro-inflammatory cytokines ↓	[89]
Angiogenesis regulation	Thalidomide	–	Murine 4T1 tumor model	Thalidomide (150 mg/kg/day, gavage)	Blood vessels formation ↓	[99]
		Cisplatin	Murine 4 T1 tumor model	Thalidomide (200 mg/kg/day, 28 days) and cisplatin (2.5 mg/kg, every other day, 28 days) intraperitoneally	Vascular normalization ↑	[100]
	Metformin	–	Murine 4T1, CT-26 and Renca tumor model	Metformin (50−300 mg/kg/day, orally, 3 weeks)	Anti-angiogenic activity ↑	[101]

–: no data. AA: arachidonic acid; AMPK: AMP-activated protein kinase; BCG: *Bacillus Calmette–Guérin*; CAFs: cancer-associated fibroblasts; CD8^+^ T cell: CD8-positive T cell; DCs: dendritic cells; ECM: extracellular matrix; E2F1: E2F transcription factor 1; FGF-2: fibroblast growth factor 2; FKBP5: FK506-binding protein 5; HA: hyaluronan; HIF-1α: hypoxia-inducible factor-1α; IFN-γ: interferon-γ; MAPK: mitogen-activated protein kinase; MDSCs: myeloid-derived suppressor cells; MICA: MHC class I polypeptide–related sequence A; MMP-9: matrix metalloproteinase-9; NK cells: natural killer cells; OVA: ovalbumin; PBMCs: peripheral blood mononuclear cells; PD-1: programmed cell death protein-1; PD-L1: programmed death-ligand 1; PDGF-B: platelet-derived growth factor-B; PlGF: placental growth factor; ROS: reactive oxygen species; TILs: tumor-infiltrating lymphocytes; Tregs: regulatory T cells; HNSCC: head and neck squamous cell carcinoma; NSCLC: non-small-cell lung cancer; ARID1A: AT-rich interactive domain-containing protein 1A.

**Table 2:** Clinical trials of FDA-approved drugs in combination with immunotherapies for cancer treatment.

Drugs	Clinical trial identifier (Phase)	Combination therapy	Targeting cancer	Status
Aspirin	NCT02659384 (Phase II)	Atezolizumab (anti-PD-L1 antibody), bevacizumab (anti-VEGF antibody)	Platinum-resistant ovarian cancer	Completed
	NCT03192059 (Phase II)	Pembrolizumab (anti-PD-1 antibody), radiotherapy, immunomodulatory cocktail (vitamin D, cyclophosphamide, curcumin and lansoprazole)	Cervical cancer, uterine cancer	Completed
	NCT03396952 (Phase II)	Pembrolizumab (anti-PD-1 antibody), ipilimumab (anti-CTLA-4 antibody)	Melanoma	Completed
	NCT03245489 (Phase I)	Pembrolizumab (anti-PD-1 antibody), clopidogrel (anti-platelet drug)	Head and neck cancer	Recruiting
	NCT03638297 (Phase II)	BAT1306 (anti-PD-1 antibody)	Colorectal cancer	Recruiting
	NCT04188119 (Phase II)	Avelumab (anti-PD-L1 antibody), lansoprazole (proton pump inhibitor)	Triple-negative breast cancer	Not yet recruiting
Celecoxib	NCT03926338 (Phase II)	Toripalimab (anti-PD-1 antibody)	dMMR/MSI-H colorectal cancer	Recruiting
	NCT03026140 (Phase II)	Ipilimumab (anti-CTLA-4 antibody), nivolumab (anti-PD-1 antibody)	Early-stage colon cancer	Recruiting
	NCT05731726 (Phase II)	Serplilumab (anti-PD-1 antibody), chemotherapy (capecitabine, oxaliplatin)	Locally advanced rectal cancer	Recruiting
	NCT05578287 (Phase II)	Tislelizumab (anti-PD-1 antibody), disitamab Vedotin (anti-HER2 ADC), chemotherapy (capecitabin)	HER2-positive metastatic colorectal cancer	Recruiting
	NCT05756166 (Phase I/II)	Pembrolizumab (anti-PD-1 antibody), chemokine modulation therapy (rintatolimod, interferon alpha-2b)	Triple-negative breast cancer	Not yet recruiting
	NCT01313429,NCT01341496,NCT02054104 (Phase I/II)	Allogenic tumor cell vaccine, chemotherapy (cyclophosphamide)	Sarcomas, melanomas, germ cell tumors, epithelial malignancies metastatic to the lungs, mediastinum, or pleura	Terminated
	NCT03864575 (Phase II)	Nivolumab (anti-PD-1 antibody)	Advanced “cold” solid tumors	Unknown
Propranolol	NCT05968690 (Phase I)	Ipilimumab (anti-CTLA-4 antibody), nivolumab (anti-PD-1 antibody), naltrexone (opioid receptor antagonist)	Advanced melanoma	Recruiting
	NCT05451043 (Phase II)	Durvalumab (anti-PD-L1 antibody), tremelimumab (anti-CTLA-4 antibody), chemotherapy drugs (gemcitabine/cisplatin/nab-paclitaxel)	Hepatocellular carcinoma, cholangiocarcinoma, pancreatic adenocarcinoma	Recruiting
	NCT05961761 (Phase II)	Pembrolizumab (anti-PD-1 antibody)	Advanced angiosarcoma, undifferentiated pleomorphic sarcoma	Recruiting
	NCT05651594 (Phase II)	Pembrolizumab (anti-PD-1 antibody), chemotherapy drugs (fluorouracil/leucovorin/oxaliplatin)	Esophageal, gastroesophageal junction adenocarcinoma	Recruiting
	NCT05741164 (Phase II)	Pembrolizumab (anti-PD-1 antibody)	Triple negative breast cancer	Not yet recruiting
Metformin	NCT04114136 (Phase II)	Nivolumab or pembrolizumab (anti-PD-1 antibody)	Solid tumor malignancies	Recruiting
	NCT03800602 (Phase II)	Nivolumab (anti-PD-1 antibody)	Metastatic colorectal cancer	Active, not recruiting
	NCT04414540 (Phase II)	Pembrolizumab (anti-PD-1 antibody)	Metastatic head and neck cancer	Active, not recruiting
	NCT05759312 (Phase I/II)	Zimberelimab (anti-PD-1 antibody)	Ovarian clear cell carcinoma	Not yet recruiting
	NCT03994744 (Phase II)	Sintilimab (anti-PD-1 antibody)	Small cell lung cancer	Unknown
	NCT03048500 (Phase II)	Nivolumab (anti-PD-1 antibody)	Non-small cell lung cancer	Unknown
Statins	NCT05636592 (Observational)	PD-1/PD-L1 inhibitors	Non-small cell lung cancer	Active, not recruiting
Cyclophosphamide	NCT00002475 (Phase II)	Tumor cell vaccine, cytokines (IFN-α, IFN-γ, GM-CSF)	Advanced cancer	Completed
	NCT01241682 (Phase I)	Tumor lysate-pulsed dendritic cells	Malignant mesothelioma	Completed
	NCT02004262,NCT01417000 (Phase II)	GVAX pancreas vaccine, CRS-207 (live, attenuated *Listeria monocytogenes*-expressing mesothelin)	Pancreatic cancer	Completed
	NCT02243371 (Phase II)	Nivolumab (anti-PD-1 antibody), GVAX pancreas vaccine, CRS-207	Pancreatic cancer	Completed
	NCT00601796 (Phase II)	Irradiated tumor cell vaccine, all-trans retinoic acid	Metastatic lung cancer	Completed
	NCT02027935 (Phase II)	Ipilimumab (anti-CTLA-4 antibody), autologous CD8^+^ Melanoma specific T Cells, aldesleukin (IL-2)	Metastatic melanoma	Completed
	NCT02423928 (Phase I)	Ipilimumab (anti-CTLA-4 antibody), autologous dendritic cells, fludarabine	Prostate cancer	Completed
	NCT01807182 (Phase II)	Tumor infiltrating lymphocytes (TILs), aldesleukin (IL-2)	Metastatic melanoma	Completed
	NCT06532812 (Phase I/II)	Tumor infiltrating lymphocytes (TILs), fludarabine	Advanced or metastatic refractory breast cancer	Recruiting
	NCT03658785 (Phase I/II)	Autologous tumor infiltrating lymphocytes (TILs), aldesleukin (IL-2), fludarabine	Advanced solid tumor	Recruiting
	NCT03971045 (Phase II)	Pembrolizumab (anti-PD-1 antibody)	Chest wall breast cancer	Not yet recruiting
	NCT03068624 (Phase I)	Autologous CD8^+^ SLC45A2-specific T lymphocytes, aldesleukin (IL-2), ipilimumab (anti-CTLA-4 antibody)	Metastatic uveal melanoma	Active, not recruiting
	NCT01420965 (Phase II)	Sipuleucel-T (therapeutic autologous vaccine), CT-011 (anti-PD-1 antibody)	Advanced prostate cancer	Terminated
	NCT01493154 (Phase I)	HPV DNA vaccine	Head and neck cancer	Terminated
	NCT02224599 (Phase I/II)	TAPA-pulsed DC vaccine, imiquimod topical cream	Progressive and/or refractory solid malignancies	Terminated
	NCT01149902 (Phase I)	Autologous immature dendritic cells, docetaxel, OK-432 (Picibanil)	Head and neck cancer	Unknown
Tadalafil	NCT03238365 (Early Phase I)	Nivolumab (anti-PD-1 antibody)	Head and neck squamous cell carcinoma	Completed
	NCT02544880 (Phase I)	Anti-tumor MUC1 vaccine	Head and neck squamous cell carcinoma	Completed
	NCT03785210 (Phase II)	Nivolumab (anti-PD-1 antibody), vancomycin	Refractory primary hepatocellular carcinoma, liver dominant metastatic cancer from colorectal cancer or pancreatic adenocarcinoma	Completed
	NCT03993353 (Phase II)	Pembrolizumab (anti-PD-1 antibody)	Recurrent or metastatic head and neck cancer	Recruiting
	NCT05014776 (Phase II)	Pembrolizumab (anti-PD-1 antibody), ipilimumab (anti-CTLA-4 antibody), CRS-207	Metastatic pancreatic cancer	Active, not recruiting
	NCT04069936 (Phase II)	Nivolumab (anti-PD-1 antibody), marrow infiltrating lymphocytes (MILs™)- NSCLC	Locally advanced and unresectable or metastatic non-small-cell lung cancer	Terminated
Thalidomide (Lenalidomide, pomalidomide)	NCT02431208 (Phase I)	Atezolizumab (anti-PD-L1 antibody), daratumumab (anti-CD-38 antibody)	Multiple myeloma	Completed
	NCT04902443 (Phase I)	Nivolumab (anti-PD-1 antibody)	Viral associated malignancies	Recruiting
	NCT01592370 (Phase I/II)	Nivolumab (anti-PD-1 antibody), ipilimumab (anti-CTLA-4 antibody), lirilumab (anti-KIR antibody), daratumumab (anti-CD-38 antibody), dexamethasone	Multiple myeloma	Active, not recruiting
	NCT03070327 (Phase I)	EGFRt/BCMA-41BBz CAR T-cell, cyclophosphamide	Multiple myeloma	Active, not recruiting
	NCT05032820 (Phase II)	BCMA CAR T-cells	Multiple myeloma	Active, not recruiting
	NCT02616640 (Phase I)	Durvalumab (anti-PD-L1 antibody), dexamethasone	Relapsed/refractory multiple myeloma	Active, not recruiting
	NCT02289222 (Phase I/II)	Pembrolizumab (anti-PD-1 antibody), dexamethasone	Relapsed/refractory multiple myeloma	Terminated
	NCT02576977 (Phase III)	Pembrolizumab (anti-PD-1 antibody), dexamethasone	Relapsed/refractory multiple myeloma	Terminated
	NCT02963610 (Phase I/II)	Pembrolizumab (anti-PD-1 antibody)	Relapsed and/or refractory solid tumors, non-small-cell lung cancer	Terminated
	NCT02807454 (Phase II)	Durvalumab (anti-PD-L1 antibody), daratumumab (anti-CD-38 antibody), dexamethasone	Relapsed/refractory multiple myeloma	Terminated
Dupilumab	NCT05013450 (Phase I/II)	PD-1/PD-L1 blocking agents	Relapsed/refractory metastatic non-small-cell lung cancer	Recruiting
	NCT06088771 (Phase I/II)	Cemiplimab (anti-PD-L1 antibody)	Early-stage resectable non-small-cell lung cancer	Recruiting
	NCT05967884 (Phase II)	Cemiplimab (anti-PD-L1 antibody)	Early-stage ER^+^ breast cancer	Not yet recruiting
	NCT03886493 (Phase II)	–	Prostate cancer	Terminated
Vitamin D	NCT06757244 (Phase II)	Dostarlimab (anti-PD-1 antibody), mFOLFIRINOX	Pancreatic cancer	Recruiting

–: no data. ADC: antibody-drug conjugate; BCMA: B-cell maturation antigen; CAR T cells: chimeric antigen receptor T cells; CRS-207: live-attenuated *Listeria monocytogenes* expressing mesothelin; CTLA-4: cytotoxic T-lymphocyte–associated protein 4; DCs: dendritic cells; dMMR: deficient mismatch repair; EGFRt: truncated epidermal growth factor receptor; ER: estrogen receptor; GM-CSF: granulocyte-macrophage colony-stimulating factor; GVAX: GM-CSF-secreting tumor cell vaccine; HER2: human epidermal growth factor receptor 2; HPV: human papillomavirus; IFN-α: interferon-α; IFN-γ: interferon-γ; IL-2: interleukin-2; KIR: killer-cell immunoglobulin-like receptor; mFOLFIRINOX: modified FOLFIRINOX (oxaliplatin, irinotecan, leucovorin, 5-fluorouracil); MILs: marrow-infiltrating lymphocytes; MSI-H: microsatellite instability-high; MUC1: mucin 1; NSCLC: non-small-cell lung cancer; PD-1: programmed cell death protein-1; PD-L1: programmed death-ligand 1; TILs: tumor-infiltrating lymphocytes; VEGF: vascular endothelial growth factor.

A central challenge lies in the pharmacological context specific to oncology. Safety profiles derived from original indications rarely extrapolate directly to cancer populations. On-target mechanisms can yield context-dependent toxicities—for example, antihypertensives provoking hypotension in otherwise normotensive patients—while the polypharmacology of many older agents introduces additional unpredictability. When combined with checkpoint inhibitors, these liabilities can be magnified, giving rise to a clinical paradox: the same mechanisms used to inflame the TME and potentiate immunotherapy may simultaneously compromise systemic immune tolerance, increasing immune-related adverse events (irAEs) [103].

Even with an appropriate mechanism and dose, the biophysical properties of solid tumors often restrict drug exposure, making delivery a persistent bottleneck. Desmoplastic stroma—characterized by collagen deposition and hyaluronan accumulation—elevates solid stress and interstitial fluid pressure, compresses intratumoral vessels, limits perfusion, and drives hypoxia; together these features impede deep penetration of both small molecules and biologics. Nanoparticle (NP) formulations have been explored to mitigate such barriers by improving solubility, leveraging the enhanced permeability and retention (EPR) effect for passive accumulation, enabling ligand-directed targeting, and permitting co-delivery of synergistic agents in calibrated ratios; nevertheless, the magnitude and consistency of the EPR effect in patients vary across tumor types; consequently, benefits are context-dependent and require careful pharmacokinetic evaluation [104].

Once delivery is addressed, patient selection becomes decisive. Single-analyte biomarkers commonly used in immuno-oncology—such as PD-L1 expression or tumor mutational burden (TMB)—capture only a fragmentary view of an evolving TME and have limited ability to predict combination benefit. A more informative direction is to develop multi-parameter, TME-centric signatures explicitly linked to mechanisms of action, integrating tissue-based immune contexture, liquid-biopsy correlates of systemic immunity, and features of the gut microbiome. The ultimate goal is to define synergy-predictive biomarkers that identify when a repurposed agent can bypass resistance pathways and sensitize tumors to immunotherapy; such signatures will require prospective validation and attention to analytical reproducibility [105].

Organoid-immune co-culture models serve as valuable tools for this purpose. By recapitulating the TME, they advance screening beyond direct cytotoxicity to evaluate non-oncological drugs that reprogram suppressive circuits and augment checkpoint inhibitor efficacy. This paradigm has identified new combinations and provided a mechanistic rationale to reposition established drugs for oncology, thereby accelerating repurposing [106]. However, model fidelity, immune repertoire representation, and cross-laboratory standardization remain variable; rigorous benchmarking and external validation are essential before routine implementation. Translating these readouts into actionable insights also requires rendering the TME both visible and computationally tractable. Spatial transcriptomics and related spatial multi-omics restore the architectural context lost during dissociation, enabling *in situ* mapping of cellular neighborhoods and suppressive pathways [107]. The resulting high-dimensional data necessitate integrative artificial intelligence (AI)/machine learning (ML) frameworks; critically, “black-box” predictions are insufficient; advancing explainable AI (XAI) is necessary to generate biologically interpretable insights that can inform testable clinical hypotheses [108].

Beyond agent selection, therapeutic sequencing significantly influences clinical outcomes. Antitumor immunity unfolds as a time-dependent cascade, and the effectiveness of combinations often depends on the sequence of administration. Certain drugs can prime the TME, conditioning it for subsequent immune activation; rational sequencing strategies that exploit such priming may enhance therapeutic efficacy, underscoring the relevance of optimal dosing schedules and chronopharmacological considerations [109]. Conventional linear trial designs are poorly suited to address these temporal dependencies. Conversely, adaptive master protocols—including basket, umbrella, and platform trials—enable concurrent testing of multiple drug–biomarker hypotheses within a single, continuously operating framework [110]. Features such as response-adaptive randomization and prospective biomarker stratification can accelerate the identification of subgroup-specific efficacy. The I-SPY 2 trial serves as a prominent example, although its broader generalizability remains a subject of ongoing investigation [111].

In conclusion, while the repurposing of drugs for cancer immunotherapy holds considerable promise, it also presents notable challenges. Overcoming these complexities necessitates a coordinated effort across researchers, clinicians, and regulatory authorities. Ultimately, through collaborative and innovative research, we can advance a new generation of personalized cancer treatments that are more effective and precisely tailored to the individual needs of patients.

## CRediT authorship contribution statement

**Yakai Song:** Writing – review & editing, Writing – original draft. **Nannan Zheng:** Writing – review & editing, Writing – original draft. **Xu Yang:** Writing – review & editing, Writing – original draft. **Zhaofan Tao:** Writing – review & editing. **Qinghui Wang:** Writing – review & editing. **Zhiyue Cao:** Writing – review & editing. **Yi Zhang:** Writing – review & editing. **Mengmeng Li:** Writing – review & editing. **Ruixin Mao:** Writing – review & editing. **Yuhao Chen:** Writing – review & editing. **Chen Zhao:** Writing – review & editing. **Huanjie Yang:** Writing – review & editing. **Bin Yang:** Writing – review & editing. **Qiuyue Ma:** Writing – review & editing. **Liangcan He:** Writing – review & editing. **Shaoqin Liu:** Writing – review & editing. **Kai Li:** Writing – review & editing, Writing – original draft, Conceptualization.

## Declaration of competing interest

The authors declare that they have no known competing financial interests or personal relationships that could have appeared to influence the work reported in this paper.
